# Influence of Different Arm Positions in the Localizer Radiograph(s) on Patient Dose during Exposure-Controlled CT Examinations of the Neck to Pelvis

**DOI:** 10.3390/tomography7030028

**Published:** 2021-07-29

**Authors:** Tony M. Svahn, Lovre Peric, Jennifer C. Ast

**Affiliations:** 1 Centre for Research and Development, Uppsala University/Region Gävleborg, 801 88 Gävle, Sweden; 2 Department of Imaging and Functional Medicine, Division Diagnostics, Gävle Hospital, 801 88 Gävle, Sweden; 3 Department of Imaging and Functional Medicine, Division Diagnostics, Hudiksvall Hospital, 824 43 Hudiksvall, Sweden; lovre0211peric@gmail.com; 4 Department of Organismal Biology, Uppsala University, 752 36 Uppsala, Sweden; ast@cleartext.se

**Keywords:** radiation dose, computed tomography, localizer radiograph, organ-based dose optimization, arm positions

## Abstract

Our aim was to examine the impact of different arm positions during imaging of the localizer radiograph(s) on effective dose for exposure-controlled computed tomography (CT) (Siemens/Canon) scans of the neck to pelvis. An anthropomorphic whole-body phantom was scanned from the neck to pelvis with the arms positioned in three different ways during the acquisition of the localizer radiograph: (i) above the head, (ii) alongside the trunk, and (iii) along the trunk with the hands placed on the abdomen. In accordance with clinical routines, the arms were not included in the subsequent helical scans. Effective doses were computed to a standard-sized patient (male/female) using a dedicated system-specific Monte Carlo-based software. Effective doses for the Canon CT scanner for the different alternatives (male/female) were (a) 5.3/6.62 mSv, (b) 5.62/7.15 mSv and (c) 5.92/7.44 mSv. For the Siemens CT scanner, effective doses were (a) 4.47/5.59 mSv, (b) 5.4/6.69 mSv and (c) 5.7/6.99 mSv. Arms placed above the head during localizer radiograph imaging in the current CT procedures substantially reduced the total effective dose to the patient.

## 1. Introduction

The increased use of computed tomography (CT) is a concern because of potential risks associated with ionizing radiation. CT scans contribute to the largest collective effective dose to patients undergoing radiological examinations, even though CT is not the most common medical imaging procedure [1], and concerns regarding this have been raised [2,3,4,5,6,7,8,9]. Optimization is necessary in order to minimize the patient dose burden while maintaining a sufficient image quality, in agreement with the ALARA (As Low As Reasonably Achievable) principle [10]. The effective radiation dose measure can be applied to quantify the radiation dose and its potential detrimental effects and is expressed in Sieverts. It estimates potential radiation hazards by taking into account the stochastic risks of cancer induction by summing organ exposure to ionizing radiation weighed against susceptibility of the specific tissues (i.e., adjusting for organs’ different sensitivity) to radiation damage. In general, the effective radiation dose measure reflects an overall risk to a body. One approach for estimating effective dose is to apply global conversion factors multiplied by the dose length product (DLP) [11]. However, these are not system-specific and do not account for manufacturer/model-specific details such as varying design in beam-shaping filter, over-ranging effects, scanner geometry, etc. In addition, in exposure-controlled CT, a single DLP does not represent the automatic exposure control (AEC) modulation pattern across the body. Manufacturers have different solutions to define image quality level in their AEC system. One approach applied by Canon is to specify the image quality level in terms of the resultant standard deviation of pixel values (SD, noise level) [12,13]. General Electric (GE) has a similar approach [12]. A lower SD value thus represents a better image quality than a higher one. Another method applied by Siemens is to set a reference mAs value for each scanner protocol that represents a reference patient [14,15]. The AEC system determines the size of the patient’s cross-section and then adapts the tube current relative to the reference mAs value [13]. In addition, angular modulation is applied, which can be adjusted in real-time considering the previous rotation, and which operates only in the xy-plane in light of the patient’s variable cross-section [15]. Notwithstanding differences in image quality definitions, all AEC systems account for the patient’s attenuation in the longitudinal direction (z-axis) based on the localizer radiograph(s) [12]. Concerning AEC modulation, one aspect is that the positioning of the arms during localizer radiograph imaging may affect the resulting dose to the patient because the modulation depends on the attenuation information of the patient in the localizer radiograph(s) [13]. Routine recommendations about arm positioning can vary among clinics. Having the arms raised during localizer radiograph(s) may result in an increase of the effective dose due to the thyroid and other relevant radiosensitive organs located above the shoulders. Consequently, it is possible that one arm positioning or the other might be more beneficial in terms of yielding an overall lower effective radiation dose. Hence, the task can be regarded as organ-based dose optimization.

The purpose of the present study was to evaluate the effect of arm position in localizer radiograph imaging on effective radiation dose for the complete CT examination of the neck to pelvis in two different CT systems.

## 2. Materials and Methods

### 2.1. Phantom Model

**Figure 1:**
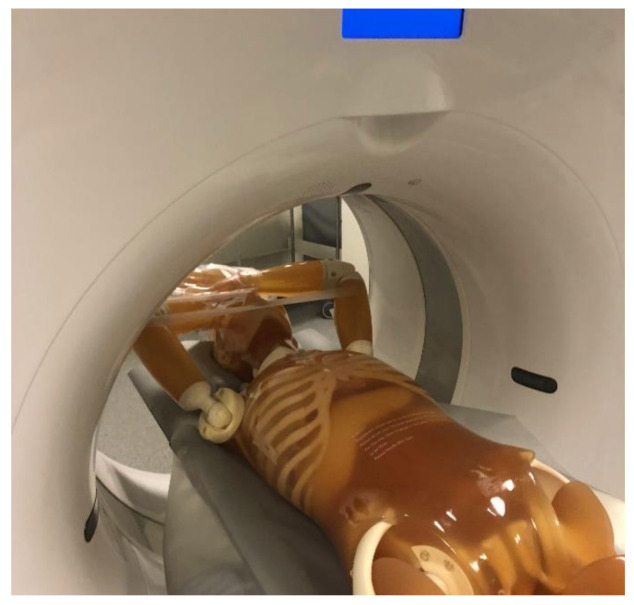
The anthropomorphic whole-body phantom (PBU−60).

A commercially available anthropomorphic whole-body phantom was used to examine various arm positions (PBU−60, Kyoto Kagaku Co. Ltd., Kyoto, Japan) [16]. The model is 165 cm long, weighs 50 kg and consists of epoxy resins and other substances to yield attenuation properties for bone structures such as the skeleton and thoracic bone structures, as well as soft-tissue organs such as the lungs, liver, mediastinum and kidneys (Figure 1).

### 2.2. CT Protocols/Image Acquisition

The whole-body phantom was scanned with two CT units (a Canon Aquilion One (Canon, Tokyo, Japan) with a frontal plus a lateral localizer radiograph and a Siemens Definition AS (Siemens, Munich, Germany) with a frontal localizer radiograph) using clinical routine CT protocols of the neck to abdomen/pelvis. The use of two localizer radiographs in Canon CT systems enables angular modulation based on attenuation information [17], while in Siemens’ CareDose 4D (Siemens, Munich, Germany), the angular modulation (the attenuation in the Lat view) is estimated from a single AP radiograph [18]. The exam protocols consist of three partially-overlapping helical scans, one mainly of the neck, one mainly of the thorax, and a final one extending from the lower parts of thorax to the pelvis (‘abdomen’). Each of these is performed with dose modulation. Three complete CT examinations were performed with the protocol having the arms/hands positioned differently during localizer radiograph imaging: (i) above the head, (ii) alongside the trunk, and (iii) alongside the trunk with hands on the abdomen. In subsequent CT scans, the arms were not included, but the patient would be asked to raise their arms during the CT scan of the chest to pelvis and to lower their arms during the CT scan of the neck.

### 2.3. Tube Out-Put

Volume CT dose index (CTDI_VOL_) and dose-length products (DLP) were extracted from dose reports and plotted for each arm position alternative (i-iii). CTDI_VOL_ and DLPs are currently not presented for Canon localizer radiographs and were subsequently obtained computationally using a Monte Carlo based software (CT-expo) (version 2.6, SASCRAD, Buchholz, Germany) [19] as described in more detail below. Tube current time products (mAs) were extracted from the Digital Imaging and Communication in Medicine (DICOM) files for individual slices of CT volumes using a macro-algorithm developed in the Image J software [20]. Information about the tube output was extracted for CT volumes reconstructed in the transverse plane for each helical scan in all three different alternatives on arm positioning. The effective mAs (mAs_eff_) was computed for each slice by dividing the mAs with the pitch factor for each type of helical scan (Table 1) and was plotted as an overlay on the AP radiographs to visualize differences in the alternatives of arm positioning.

### 2.4. Radiation Dose

A dedicated Monte Carlo (MC)-based program was used for computing effective dose (CT-expo) (version 2.6, SASCRAD, Buchholz, Germany) [19] using the resulting CT scan parameters as an in-put (Table 1). The computations include organs at risk in the field of view (FOV) in addition to scatter contributions to organs outside the FOV. In CT-expo, dose estimates are scanner-specific, accounting for beam shape, filter, voltage, geometry, over-scanning and beaming; these estimates were applied to the helical scans well as the CT scouts. The effective dose computations are based on reference persons as recommended by the international Commission on Radiological Protection (ICRP) [21]. The tissue weights established by ICRP in 2007 (i.e., ICRP 103) were used [21]. 

### 2.5. Statistical Analysis

Paired samples *t*-tests were performed using the Prism software version 6.0 (GraphPad software, Inc., La Jolia, CA, USA) to examine whether there were differences in computed individual effective doses for the different scenarios of arm positioning. Differences were considered statistically significant if the alpha was below 0.05.

## 3. Results

### 3.1. CTDI_VOL_ and DLPs

**Figure 2:**
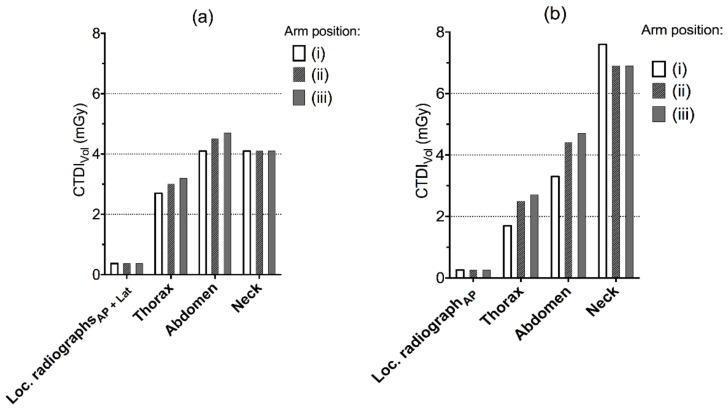
Volume CT dose index (CTDI_VOL_) by helical scan for different scenarios of arm positioning in the CT localizer radiographs ((i), arms above head, (ii) arms along the trunk and (iii) arms along the trunk with hands placed on the abdomen) for (**a**) Canon Aquilion One and (**b**) Siemens Definition AS.

**Figure 3:**
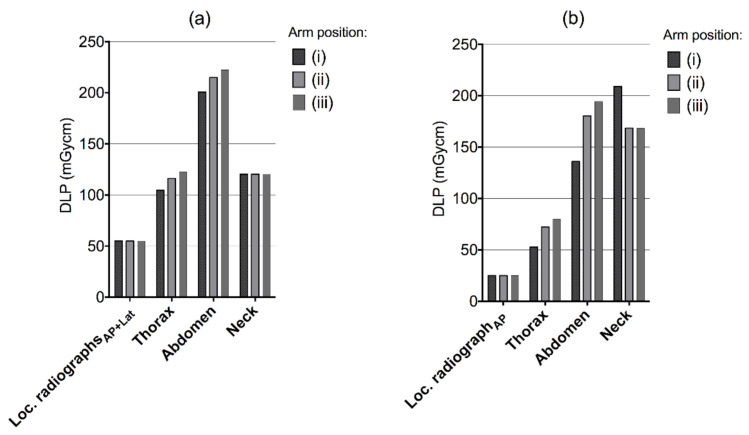
Dose-length products by helical scan for different scenarios of arm positioning in the CT localizer radiographs ((i), arms above head, (ii) arms along the trunk and (iii) arms along the trunk with hands placed on the abdomen) for (**a**) Canon Aquilion One and (**b**) Siemens Definition AS.

There were increases in CTDI_VOL_ and DLP to the thorax and abdominal region when the arms were placed alongside the trunk (position ii) relative to having the arms raised (position i) (Figure 2 and Figure 3). For the helical scan of the neck, the values were higher with the arms raised for the Siemens CT system, while they were the same for the Canon system regardless of arm positioning. Having the arms raised (i) in comparison to (ii) arms alongside the trunk or (iii) alongside the trunk with hands on the abdomen yielded a decreased DLP for the complete examination with 25.8 and 39.5 mGycm for Canon, and 23.3 and 44.6 mGycm for Siemens.

### 3.2. Effective mAs

#### 3.2.1. Canon’s Protocol

**Figure 4:**
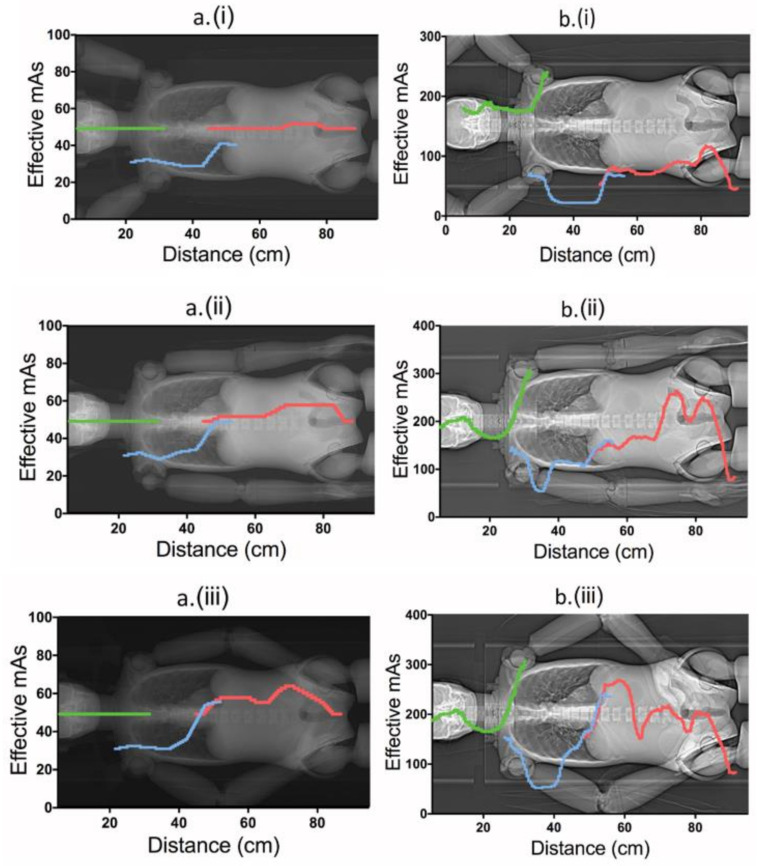
Effective mAs for the three different helical scans in different scenarios of arm positioning in the localizer radiographs (using corresponding protocol at both CT systems): (i) arms raised above the head, (ii) arms lowered along the trunk and (iii) arms along the trunk with hands placed on the abdomen, for Canon Aquilion One ((**a**); left column) and Siemens Definition AS ((**b**); right column).

Having the arms raised (position i) resulted in a constant effective mAs (mAs_eff_) for the neck scan (Figure 4a (i), green) and was almost constant for the abdomen-pelvis scan (Figure 4a (iii), red), i.e., due to constraints of a minimum mA before applying tube-current modulation (Table 1). When the arms were positioned alongside the body (ii), mAs_eff_ increased gradually for the thorax to pelvis scans, while remaining constant for the neck scan. Having the arms bent with hands placed on the abdomen yielded further elevated mAs_eff_ values (Figure 4a (ii vs. iii); at a distance 40–60 cm; blue/red-coloured curves). The mAs_eff_ was constant for the neck scan here as well.

#### 3.2.2. Siemens’ Protocol

In contrast to the Canon protocol, the Siemens tube current modulated in all three helical scans for all alternatives of arm positions, with elevated mAs_eff_ for the neck (Figure 4b (i vs. ii and iii)). Overall, the mAs_eff_ was further increased for positions (ii) and (iii) compared to (i) (Figure 4b). Similar to Canon, the mAs_eff_ was higher for Siemens for position (iii) relative to (ii) when the arms were bent (at distance 40–60 cm). Having the hands placed on the abdomen yielded slightly lower mAs_eff_ than having them alongside the trunk (iii vs. ii, at distance 70–90 cm, red). 

### 3.3. Effective Dose

**Figure 5:**
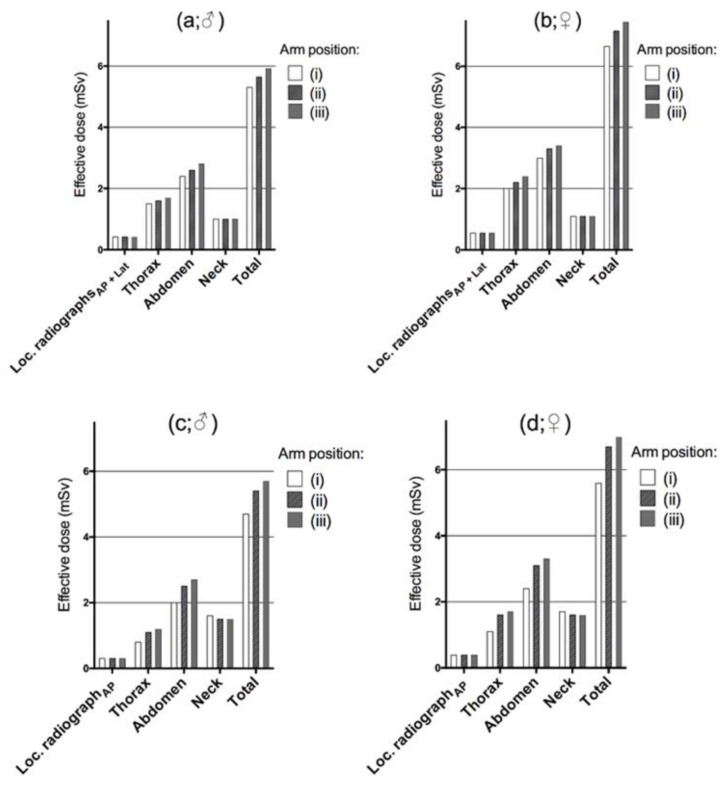
Effective doses (mSv) as a result of different positioning of the arms during imaging of the localizer radiograph(s) (Figure 2; i–iii). Results are shown for males (**a**,**c**) and females (**b**,**d**) for the different CT systems (Canon a, b and Siemens c, d) and anatomical regions.

With the Canon CT unit, there was a corresponding increase in effective dose for the complete examination of 6% for males and 8% for females for arm position (ii), and 11.5% and 12.3% respectively for the alternative (iii) compared to (i) (Figure 5), which were statistically significant (ii vs. i, *p* = 0.02; iii vs. i, *p* = 0.01). With the Siemens CT, there was a significant relative increase in patient dose for the complete examination for males and females by 15% and 20%, respectively, when the arms were positioned alongside the abdomen (Figure 5 (ii)) compared to having the arms raised above the head (Figure 5 (i); *p* = 0.02) during imaging of the localizer radiographs. Patient dose increased by 21% and 25% when the hands were placed on the abdomen compared to arms raised (iii relative i, Figure 5; *p* = 0.01). For the Siemens CT examination, the effective dose was higher for the neck region when the arms were placed above the head, while for the Canon CT scanner, the dose at the neck region remained the same for all alternatives as a result of the constant tube output (i–iii). 

## 4. Discussion

The principle of ALARA (“as low as reasonably achievable”) introduced by the International Commission of Radiological Protection (DLP) has become more relevant in this era of increased use of CT for diagnostic and interventional procedures, and efforts should be made to reduce unnecessary radiation exposure from CT [10]. A clinical CT examination often covers a range of different anatomical regions that have tissues with variable attenuation values. Automatic Exposure Control (AEC) systems account for a patient’s shape, size, and attenuation with the purpose of increasing consistency in image quality among patients, partially or completely based on the information derived from the localizer radiographs. If an AEC system is not used, it is difficult to assess an adequate image quality/dose level [22]. A subsequent risk is, for instance, that smaller patients may receive higher doses of radiation than necessary, or that the image quality for larger patients may be hampered by increased noise.

In the present work, we applied the most recent Monte-Carlo-based methods (vendor-specific, accounting for details previously described, under the ‘Radiation dose’ paragraph) to estimate the complete effective radiation dose to patients undergoing CT exams at two different systems for various scenarios of arm positioning [19,23]. The task itself, organ-based optimization, is difficult, since it is a matter of counterbalance. One scenario (e.g., arms raised) might increase the dose primarily to the thyroid, but not to higher-risk organs below the shoulders, while different scenarios may increase the dose to multiple higher-risk organs below the shoulders, but not those above the shoulders.

The main result showed that the effective radiation dose of the complete examination increased when the arms were positioned alongside the trunk (ii) and increased further when the hands were placed on the abdomen (alternative iii) during localizer radiograph imaging in comparison to having the arms raised above the head (alternative i). For alternatives ii and iii, attenuation derived from the localizer radiograph represent a ‘larger patient’ below the shoulders, and subsequently the mAs_eff_ increased to these regions (Figure 2). Even though the dose to the thyroid increased with arms raised for the Siemens system (Figure 2), the overall effective dose to the patient was reduced, which was true for both systems. Physically, the arms are not completely included in the localizer radiograph(s) when the arms are raised, which means that the excluded parts cannot contribute to any additional attenuation.

A relevant observation was that for both CT systems, the effective mAs went up when the arms were bent (Figure 2; alternative iii vs. ii around the distance 40 to 60 cm). The reason for this is that the arms are placed at an angle (Figure 2), which means that the radiation has a longer travel path through the arms in the lateral direction. Having the hands placed on the abdomen also increases the photon travel path in the AP direction (Figure 2). As noted in the Canon protocol, there was a dose modulation constraint of 80 mA resulting in a fixed mAs value for the neck region as well as the abdominal region (Table 1), which limits the tube load to become to lower than ~50 mAs for leaner patients (Figure 2). As such, for the neck region, having the arms raised did not cause a sufficiently low noise level to further elevate mAs (or the dose) to the thyroid (Figure 2 (i–iii); left column). If contrast media is being applied, it is important that the image quality is sufficient for detection of the media. Therefore, in both CT systems, the modulations along the neck region are set at a relatively higher image quality/lower noise level in comparison to the thorax scan. 

The effect seen on effective dose was relatively larger for the Siemens CT unit. Canon’s mA constraints also have implications for the dose differences seen (i–iii). For larger patients, when constraints are surpassed, the differences will likely become larger because there are more radiosensitive organs below the shoulders that will be exposed.

The relative dose differences should be even higher for larger patients, or, specifically, for patients with larger arms in relation to their body than the phantom model. As shown for the Siemens system, there was an effective dose increase apparent in the neck region. However, the dose is further increased to other at-risk organs when the arms are placed along the chest or trunk. In practice, dose modulation could also result in a higher percentage output over the breasts for females (because the breast tissue increases the attenuation). Besides the general dose modulation techniques (i.e., CareDose 4D and SureExposure), organ-based dose modulation (ODM) may further help to reduce radiation exposure to sensitive organs [24,25]. For females, the breasts and the ovaries are especially sensitive to radiation, which tends to result in higher effective doses than for males (Figure 5).

It is known that there are differences between manufacturers in how dose modulation works. Canon’s CT system uses an image quality reference to keep a constant image noise level (standard deviation value) regardless of attenuation level, using tube currents within prescribed minimum and maximum values. These can be set to ensure a certain level of image quality. In the current case for the neck scan, high image quality is important for early detection of the contrast media, if applicable. Siemens, on the other hand applies a quality reference mAs, thereby striving to keep a constant image quality regardless of attenuation level, with a reference to a target mAs level for a standard-sized patient. In the image quality references in both systems, it is acknowledged that larger patients have larger amounts of visceral fat, which increases the contrast within the body (i.e., SureExposure 3D Adaptive and CareDose 4D). As such it leads to a lower rate of dose increase for larger/fattier patients compared to smaller/leaner patients [18]. Fundamentally, all AEC systems account for the patients’ attenuation in the longitudinal direction (z-axis) based on the localizer radiograph(s) [12]. With this fact in mind, and because most at-risk organs are situated below the shoulders, it is unlikely that the results of other systems would be much different in terms of providing different ordering of the results (i.e., highest dose, alternative: iii > ii > i). It seems that having the arms along the trunk corresponds to a ‘larger patient’ over multiple radiosensitive organs. While a complete (x, y, z) real-time adjusted AEC in theory might reduce the necessity for a localizer radiograph, current real-time AEC only applies in the angular modulation (i.e., in Care Dose 4D) and the localizer radiograph is utilized to set the parameters in the longitudinal direction (z) [15]. To achieve a stable dose modulation, the information from the localizer radiograph about the z-axis is used in the longitudinal direction [13], although it is clear that the magnitude of the impact may vary depending on technical factors, as seen in our results.

The current study has some limitations. With respect to generalizability, the effective dose is known to vary for individuals [22], which for instance depends on differences in patient sizes and organs [22]. In the MC estimates, however, data was projected to standard-sized patients to find out what scenario yields the lowest patient dose. Therefore, the relative effects seen should be valid for the general patient population. With regards to repeatability, we did not perform additional measures to evaluate consistency in the dose modulation because it has recently been tested thoroughly, and variability among repeated CT scans with the same settings/parameters were found to be negligible [26].

## 5. Conclusions

In summary, in the present work, we examined different scenarios of arm positioning on effective dose on two different CT systems and found that having the patients’ arms raised during scout imaging substantially reduced the total effective dose to the patient shown in the exposure parameters (mAs_eff_), tube outputs, dose-length products, and in the effective radiation dose. In addition, as indicated in mAs_eff_, the arms should preferentially be stretched out straight (unbent, parallel to the table travel direction) to further minimize the attenuation, tube output and, hence, the radiation dose to the patient.

## Figures and Tables

**Table 1:** CT scanning parameters.

Parameters	Canon Aquilion One *^, §^	Siemens Definition AS ^£, §^
Beam collimation (mm)	80 × 0.5	64 × 0.6
Scanning mode	Helical	Helical
Pitch [neck]	0.813	0.8
Pitch [thorax]	1.388	0.5
Pitch [abdomen]	0.813	0.6
Rotation time	0.5	0.35
Tube voltage (kVp)	120	100
AEC	SureExposure 3D	Care Dose 4D
Scanning direction	Head first	Head first

* A minimum of 80 mA is set in the dose modulation to ascertain a sufficient quality for the neck and thorax scans. ^£^ A ref mAs of 115 is used in the neck scan, 100 in the thorax scan, and 110 in the abdominal scan. ^§^ Reconstructions are performed in the transverse plane (3 mm slice thickness).

## Data Availability

Not applicable.
